# Corneal perforation after corneal foreign body - Case Report

**DOI:** 10.22336/rjo.2023.14

**Published:** 2023

**Authors:** Sorin Simion Macarie, Fodor Vlad, Daniela Mariana Macarie

**Affiliations:** * “Iuliu Hațieganu” University of Medicine and Pharmacy, Cluj-Napoca, Romania; ** Cluj County Emergency Hospital, Cluj-Napoca, Romania; *** A.I. S.C.B.I. Cluj, Cluj-Napoca, Romania

**Keywords:** corneal melting, corneal anaesthesia, neurotrophic keratitis, corneal foreign body, corneal perforation

## Abstract

**Purpose:** To present the case of a patient with a history of trauma and corneal foreign body in the right eye, followed by decreased visual acuity in the right eye, corneal perforation with good recovery after surgical treatment.

**Material and method:** We report a case of a patient who presented to our clinic with a sudden decrease of visual acuity in the right eye, two months after an incident resulting in a corneal foreign body in the right eye. In the case presented, the patient applied a local medical self-treatment, an antibiotic and a topical corticosteroid. After a few weeks, the patient presented to the ophthalmologist, a foreign body was extracted from the cornea of the right eye and a topical treatment with a non-steroidal anti-inflammatory drug, a cycloplegic and an antibiotic were indicated. However, corneal perforation occurred and the patient was urgently sent to our service, where a corneal anaesthesia was also found.

**Results:** Corneal perforation healed with a minor paracentral opacification.

**Discussions:** Corneal perforation in our patient was due to corneal melting because of topical steroid anti-inflammatory autotherapy, late corneal foreign body extraction and topical treatment with non-steroidal anti-inflammatory drugs. Corneal anesthesia is also an important factor that enhances corneal melting and perforation. The surgical intervention performed healed the corneal perforation.

**Conclusions:** Corneal anaesthesia and topical anti-inflammatory administration led to corneal perforation. Corneal sensitivity should be tested in patients with corneal foreign body. Corneal patching proved to be an adequate solution in this patient.

## Introduction

Corneal foreign bodies are frequently encountered in ophthalmic practice. In most cases, after their extraction, the patient’s evolution is favorable, with rapid corneal healing, the appearance of a discrete corneal scar and total or partial recovery of visual acuity (in the case of central corneal foreign bodies). Certain circumstances (e.g. late presentation to the doctor, self-medication, topical application of corticosteroids or non-steroidal anti-inflammatory drugs, corneal anaesthesia or hypoesthesia) may lead to an unfavorable outcome, characterized by late or absence of corneal epithelialization, corneal melting and corneal perforation. Under the circumstances, the consequences on vision may be serious.

## Case report

We present the case of a 45-year-old patient with a sudden and painless decrease in visual acuity in the right eye, without other symptoms, two months after an ocular trauma resulting in a corneal metal foreign body after handling a metal cutting machine, without the use of appropriate eye protection equipment. After this incident, the patient presented a discrete ocular symptomatology (discrete discomfort), and self-administered topical medication to the right eye (antibiotic and corticosteroid). Two weeks after the end of this treatment, the persistence of a small local discomfort determined the patient to present to the territorial ophthalmology service, where the corneal foreign body was extracted and local treatment with antibiotic and corneal re-epithelialization was recommended. Six weeks after the initial event, the patient presented again to the ophthalmologist in the territory, where he tried to extract the remaining rust from the cornea and finally applied a therapeutic lens with local treatment recommendations (Vigamox, Indocollyre, Tropicamide). The evolution was still unfavorable, and corneal perforation occurred, which is why the patient was sent to the Ophthalmology Clinic in Cluj-Napoca, where the existence of a paracentral inferior corneal perforation of about 2.5 mm diameter, with the iris trapped in the perforation and the presence of a therapeutic contact lens were found.

**Fig. 1:**
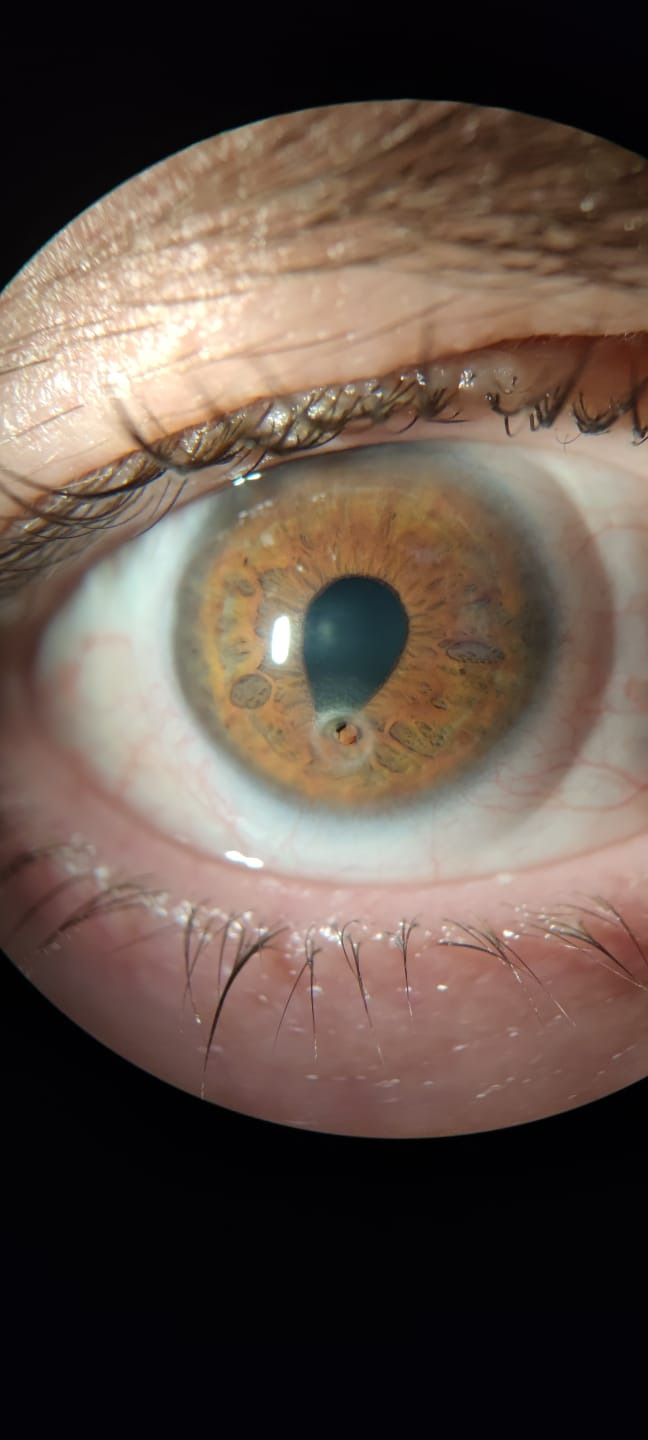
Anterior pole of right eye at admission in the hospital

Vision evaluation showed a visual acuity of 0.2 without correction in the right eye and 0.6 with pinhole in the right eye and 1 with pinhole in the left eye. The left eye showed a hyperopia of +2.00 D. The right eye was hypotonic at digital palpation. Intraocular pressure in the left eye was 17 mmHg.

The examination of the anterior segment of the right eye revealed a very mild conjunctival congestion, a hypontransparent ring-shaped corneal lesion of about 3.5 mm diameter with a corneal perforation of about 2.5 mm present paracentrally inferior, anterior chamber absent, pupil vertically oval deformed, iris herniated through the corneal perforation, transparent lens, and therapeutic contact lens present. Left eye anterior pole an both eyes fundus examination revealed normal relations. 

Corneal sensitivity testing revealed corneal anesthesia in the right eye and very marked hypoesthesia in the left eye. In this context, the patient was questioned about toxic consumption and he reported a daily consumption of small amounts of alcohol.

Among the laboratory tests performed, dyslipidemia, GammaGT and ferritin above the normal values were noted.

The decision was made to perform surgery and a scleral autograft was sutured to the corneal perforation. Postoperatively, a therapeutic contact lens was applied. The evolution was favorable, the anterior chamber returned to normal depth, the aqueous humor was clear. At discharge, visual acuity of the right eye was 0.8 with correction, anterior pole right eye scleral graft present, cornea otherwise clear, anterior chamber normal depth, round pupil, therapeutic contact lens, normal intraocular tonus in both eyes.

**Fig. 2:**
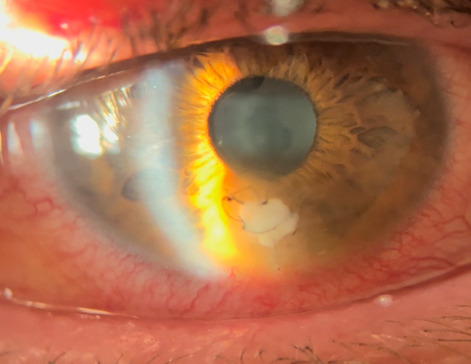
Anterior pole right eye at 3 days after surgery

**Fig. 2** shows the appearance of the right eye at 3 days postoperatively. At 14 days postoperatively, the visual acuity of the right eye was 1 with correction.

**Fig. 3:**
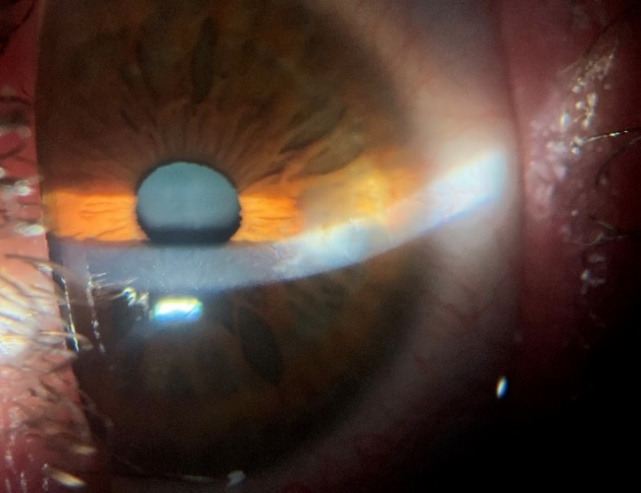
Anterior pole right eye at 3 months after surgery

At 6 weeks postoperatively, the aspect was stationary and the therapeutic contact lens was removed. At 2 months postoperatively, it was decided to remove two of the sutures and after another 2 weeks the remaining sutures were removed. **Fig. 3** shows the appearance at 3 months postoperatively. The scleral graft was seen to be integrated and less opaque than in the early postoperative days. At 3 months, visual acuity of the right eye = 1 with correction +0.75 D cyl axis 85°.

## Results

Corneal perforation healed with a minor paracentral opacification. Visual acuity at 3 months after surgery was 1 with a minor correction (+0,75 D cyl axis 85°).

## Discussions

The unfavorable evolution of the patient after the trauma resulting in the existence of a corneal foreign body was due to several factors: late presentation to the ophthalmologist, local self-therapy with steroidal anti-inflammatory, corneal anesthesia and local therapy with non-steroidal anti-inflammatory drugs.

Corneal anesthesia causes very discrete or even the absence of symptoms in patients with corneal disorders, including corneal foreign bodies, leading to late presentation to the doctor. Corneal esthesiometry is rarely performed in patients with corneal foreign bodies, so therapeutic management may be inadequate. The causes of corneal anesthesia are diverse (age, diabetes mellitus, anterior pole surgery - including refractive surgery, corneal trauma, viral keratitis, etc.) [**1**], damage to corneal sensory nerve endings causing slowing or even abolition of the regeneration processes, alteration of corneal metabolism and trophicity, and lack of reflex response to noxious stimuli. The incidence of neurotrophic keratopathy is low (less than 0.05%) [**2**], but it is underestimated because of the less frequent corneal sensitivity testing.

In addition, topical therapy with steroidal and non-steroidal anti-inflammatory drugs produces the phenomenon of corneal melting, favoured by corneal anaesthesia or hypoesthesia [**3**-**6**]. The literature reports patients with neurotrophic keratitis induced by topical treatment with steroidal or non-steroidal anti-inflammatory drugs [**6**,**7**].

Restoration of the structural integrity of the eyeball following a perforating corneal defect must be performed quickly, scleral tissue autograft being a method that is easy and rapidly available [**8**,**9**]. In our case, the visual functional recovery was good because the perforation was relatively far from the centre of the cornea. Aesthetic deficit is attenuated over time [**9**]. In the rare cases in which corneal perforation occurs, there are therapeutic alternatives such as hot keratoplasty and application of amniotic membrane associated with conjunctival flap cover (Gunderson). However, corneal grafting and even amniotic membrane are not available at short time. Therapeutic contact lenses have little efficacy in healing perforation, but can prevent infectious complications. Cacicol drops should be a good alternative, but the product has been withdrawn.

In the case of central corneal perforations, the scleral autograft allows preservation of the eyeball for a subsequent keratoplasty that will improve visual function.

## Conclusions

In conclusion, scleral autograft is an effective and easily accessible therapeutic option in situations of corneal perforation, effectively restoring the integrity of the eyeball and preventing possible serious complications. The functional outcome is good in cases of peripheral or paracentral corneal perforations. Corneal esthesiometry should be routinely performed in patients with corneal microtrauma and corneal foreign bodies. Corneal anesthesia together with topical use of anti-inflammatory agents may induce progression to corneal perforation in these patients.

**Conflict of Interest Statement**

The authors declare that there are no conflicts of interest.

**Informed Consent and Human and Animal Rights statement**

An informed consent was obtained from the patient included in the study.

**Authorization for the use of human subjects**

Ethical approval: The research related to human use complies with all the relevant national regulations, institutional policies, it is in accordance with the tenets of the Helsinki Declaration and has been approved by the review board of Cluj County Emergency Hospital, Cluj-Napoca, Romania.

**Acknowledgements**

None.

**Sources of Funding**

None.

**Disclosures**

None.
